# Estradiol pretreatment in GnRH antagonist protocol for IVF/ICSI treatment

**DOI:** 10.1515/med-2022-0594

**Published:** 2022-11-21

**Authors:** Shaomi Zhu, Zhexi Lv, Linjiang Song, Qinxiu Zhang, Yiyue Fan, Junjun Li

**Affiliations:** School of Medical and Life Sciences/Reproductive & Women-Children Hospital, Chengdu University of Traditional Chinese Medicine, Chengdu, 611137, Sichuan Province, China; Chengdu Fifth People’s Hospital/The Fifth People’s Hospital of Chengdu University of Traditional Chinese Medicine, No. 33, Mashi Street, Wenjiang District, Chengdu, 611130, Sichuan Province, China; School of Medical and Life Sciences/Reproductive & Women-Children Hospital, Chengdu University of Traditional Chinese Medicine, No. 1166 Liutai Avenue, Wenjiang District, Chengdu, 611137, Sichuan Province, China

**Keywords:** estradiol, GnRH antagonist, IVF/ICSI, meta-analysis

## Abstract

We conducted a systematic review and meta-analysis of all published data to determine the impact of estradiol pretreatment on reproductive outcomes and ovary stimulation characteristics for *in vitro* fertilization (IVF)/intracytoplasmic sperm injection (ICSI) treatment with gonadotropin-releasing hormone (GnRH) antagonist protocol. MEDLINE, EMBASE, Cochrane Library, Web of Science, and China National Knowledge Infrastructure were searched, and any randomized controlled trials associated with estradiol pretreatment in GnRH antagonist protocol were included. Seven studies (1,236 patients) were included in the present study. The pooled data from the meta-analysis demonstrated no significant difference in ongoing pregnancy rate (odds ratio (OR): 0.92 (95% CI: 0.69–1.21; *P* = 0.53) and live birth rate OR: 0.98 (95% CI: 0.74–1.30; *P* = 0.90) between patients with and those without estradiol pretreatment in GnRH antagonist protocol. Duration of gonadotropin exposure, gonadotropin consumption, and the number of cumulus–oocyte complexes were not significantly different between groups. Luteal estradiol pretreatment in IVF/ICSI cycles with GnRH antagonist protocol in normal ovary responding population does not affect the reproductive outcomes. It is an encouraging option to facilitate cycle scheduling in GnRH antagonist protocol, for luteal estradiol pretreatment does not increase the duration of gonadotropin exposure or gonadotropin consumption.

## 1 Introduction

In recent years, gonadotropin-releasing hormone (GnRH) antagonist protocol is widely used for *in vitro* fertilization (IVF)/intracytoplasmic sperm injection (ICSI) treatment due to its simplicity, safety, and effectiveness with the proportion increasing from 6% in 2014 to 37% in 2021 [1]. Compared with the long GnRH agonist protocol, GnRH antagonist protocol offers similar pregnancy rates and definite advantages, including absence of possible ovarian cyst and peri-menopausal symptoms caused by pituitary desensitization, shorter treatment duration, and a lower consumption of gonadotropin, which may decrease the risk of ovarian hyper-stimulation syndrome in patients with high ovarian response [2,3]. However, in the use of GnRH antagonist protocol, there are still some problems remaining to be solved. The biggest concern is antral follicular synchronization. Steroid pretreatment can regulate the sex hormone negative feedback via pituitary–hypothalamus and synchronize the follicular cohort before controlled ovulation stimulation (COH). Therefore, a lot of attention has been paid to the potential benefits of steroid pretreatment in GnRH antagonist protocol.

Some studies have researched on the pretreatment with oral contraceptive pills (OCs) containing estrogen plus progestin, especially for patients who did not get pregnant after surgery treatment for endometriosis and finally selected IVF/ICSI to help solve fertility problems [4,5]. However, several research including two recent studies [6,7] reported a significant decrease of ongoing pregnancy rate, which was probably caused by the negative effect of high-potency progestin on endometrial receptivity. After initial exploration, the current focus in clinical practice has gradually shifted to improving and optimizing the GnRH antagonist protocol. In recent years, an issue highly concerned is the possibility of luteal estradiol pretreatment in GnRH antagonist protocol. Compared with OCs, estradiol pretreatment produces a milder suppression of follicle stimulating hormone (FSH), which probably results in a lesser reduction of antral follicle diameter. This opens the possibility of improving the response of ovary to gonadotropin in patients with size-variable antral follicles. Indeed, previous studies have shown that estradiol pretreatment are effective in improving follicle synchronization within the cohort and enhancing oocytes recovery [8]. We have demonstrated that luteal FSH suppression achieved by the administration of estradiol is effective to synchronize follicle growth by minimizing their mean diameter before COH. However, the available studies have not been powerful enough to evaluate the impact of luteal estradiol administration on pregnancy outcomes and COH characteristics (consumption of gonadotropin and duration of gonadotropin exposure). The effect of cycle pretreatment with estradiol alone on pregnancy outcomes is yet poorly known. The data of many high quality randomized controlled trials (RCTs) can be pooled in a systematic review and may get a more reliable answer associated with luteal estradiol administration in GnRH antagonist protocol. Based on the above considerations, the objective of the present meta-analysis was to determine the effects of estradiol pretreatment on pregnancy outcomes and COH characteristics in patients treated with GnRH antagonist protocol in IVF/ICSI cycles.

## 2 Methods

### 2.1 Systematic search and strategy

**Figure 1:**
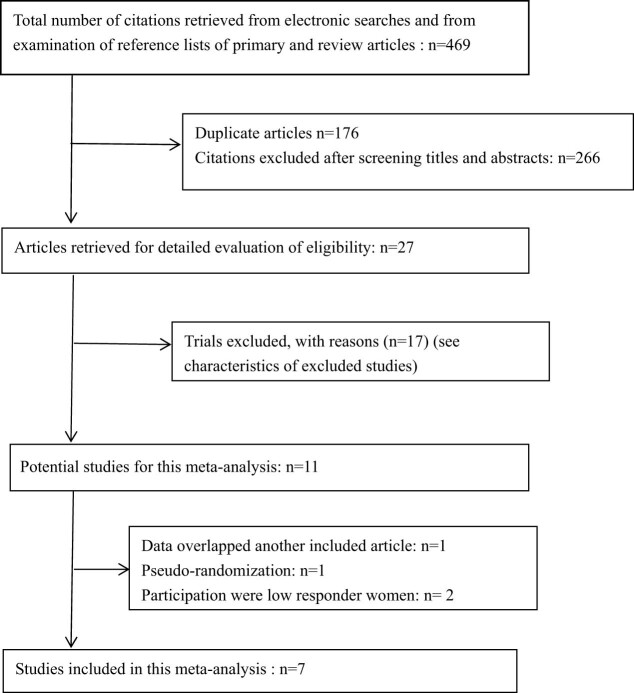
Flow chart of the study selection process used for systematic review and meta-analysis.

After the research plan was established, from May 2021 to September 2021, a literature search was conducted strictly restricted to RCTs without language restrictions. We searched the official home pages of EMBASE, Web of science, MEDLINE, Cochrane Library, and China National Knowledge Infrastructure for the related studies about the effect of luteal estradiol pretreatment for COH in patients undergoing IVF/ICSI with GnRH-antagonist protocol. The following search strategy was used: (“estrogen” or “estradiol”) AND (“GnRH antagonist”) AND (“assisted reproductive techniques (ART)” or “ART” or “IVF” or “*in vitro* fertilization” or “ICSI” or “intracytoplasmic sperm injections”) AND (“randomized controlled trial” or “clinical study” or “multicenter study” or “double blind procedure” or “single blind trial”). The references of retrieved literature were also searched to identify another potential research (Figure 1).

### 2.2 Inclusion and exclusion criteria

Inclusion and exclusion criteria of the studies were established before the literature search. The inclusion criteria were defined as follows: (i) prospective RCT design; (ii) COH with GnRH antagonist protocol; (iii) regular ovulatory menstrual cycles every 25–35 days; (iv) both ovaries present and body mass indexes (BMI) ranging between 18 and 30 kg/m^2^; less than three previous unsuccessful IVF/ICSI cycles. Eligible studies should contain sufficient information to evaluate whether randomization was standard and whether the baseline demographic characteristics, ovarian stimulation protocols, and the total number of embryos transferred were comparable between groups. Research that did not meet the above criteria would be excluded. Every effort was made to contact the authors to obtain the data not available in the literature, when necessary. Selection of studies and evaluation of methodological quality were accomplished by two of the investigators.

### 2.3 Data extraction

The following data were recorded from the included studies independently by two of the investigators: first author, time of publication, demographic characteristics, procedural characteristics (inclusion and exclusion criteria, number of patients included, type of gonadotropin administered, criteria for final oocyte maturation triggering in ART cycles, duration between oocyte retrieval and fertilization, method of fertilization, the day of embryo transfer, and medication for luteal phase support), and outcome data (number of oocytes retrieved, consumption of gonadotropin, duration of gonadotropin exposure, ongoing pregnancy, and live birth rate). Any disagreement on the included data between the investigators was solved by discussion. When outcome indicators were not clearly described in the studies meeting the inclusion criteria, the information was collected by converting the published data or contacting the corresponding author.

### 2.4 Types of outcome measures

Selected primary outcome measures were the ongoing pregnancy rate per cycle (defined as the presence of the embryo heartbeat by ultrasound examination at 12 weeks of gestation), spontaneous abortion rate (defined as miscarriage before 20 weeks of gestation), and live birth rate (after 20 weeks of gestation, at least one surviving newborn is defined as a live birth) [9]. When the reproductive outcomes of dropped-out patients were not available, the corresponding authors would be contacted. If the data were still inaccessible, these dropped outs would be analyzed as not pregnant. Secondary outcome measures were duration of ovarian stimulation, consumption of gonadotropin, and number of oocytes retrieved.

### 2.5 Quantitative analysis

The present statistical analysis was performed with the RevMan software (Version 5.0, Cochrane Collaboration). Continuous variables were analyzed by means of weighted mean difference (WMD) with 95% confidence interval (CI). Dichotomous data were expressed as odds ratio (OR) with 95% CI. The study-to-study variation was evaluated by the Cochrane’s *Q*-test. A fixed-effects model was performed when no heterogeneity was present. A random-effects model was performed when significant heterogeneity was present. An intention to treat analysis was applied in randomized trials with missing outcome data.

### 2.6 Risk of bias assessment

**Figure 2:**
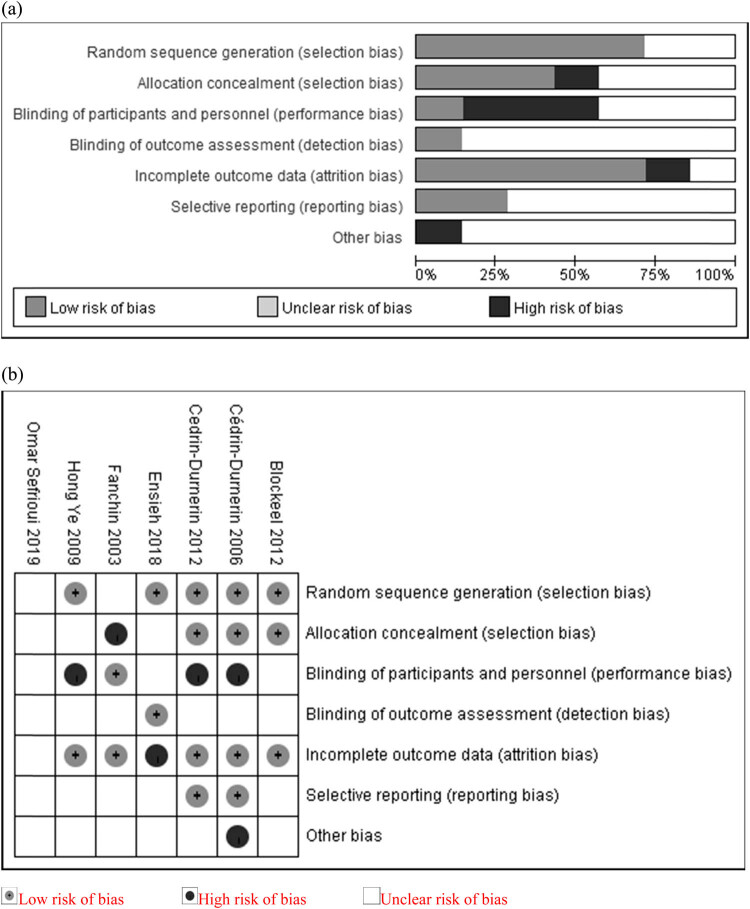
Risk of bias for included trials: (a) risk of bias graph and (b) risk of bias summary.

Cochrane Collaboration tool [10] was used to evaluate the risk of bias for the RCTs included (Figure 2). Two investigators independently evaluated the quality of the studies. References of previous published meta-analysis that met the enrollment criteria were included for pooled analysis. When discrepancies occurred, a third investigator was consulted to reach a consensus. The current study was approved by the Ethics Committee of Affiliated Reproductive & Women-Children Hospital, Chengdu University of Traditional Chinese Medicine (Approval document No. 2020-03).

## 3 Results

### 3.1 Systematic review

Since abstract publications could not supply detailed information for methodological quality and data synthesis, the studies published in abstract form were excluded in the present study. A total of 469 papers were eligible for inclusion in the study, 27 full-text versions of articles were reviewed. Seven of them fulfilled all inclusion criteria and no exclusion criteria [11–17]. Thus, the seven articles were included in the current analysis without disagreement between the investigators responsible for literature search (Figure 1). The included trials were of high quality according to the modified Jadad scale (Jadad score ≥3) [18]. Further information about these studies is given in Tables 1–3. A total of 1,236 patients were randomized to receive either estradiol pretreatment (*n* = 617) or no pretreatment (*n* = 619) before ovarian stimulation. Two trials were multicentric. All seven publications used 17β-estradiol 4 mg/day and the duration of administration ranged between 6 and 15 days. Recombinant FSH was used for COH in all the seven studies, and the patients were stimulated with a fixed GnRH antagonist protocol in two RCTs. Type of fertilization included both IVF and ICSI. In all seven trials, transvaginal progesterone was used for luteal phase support.

**Table 1:** Clinical characteristics of included trials

Authors, year, country of origin, journal	Multicenter	Study period	Patients/Allocation	Randomization method	Allocation concealment	Primary outcome
Fanchin et al., 2003, France, Hum Reprod	No	2003	90 (47 estradiol, 43 control)	Table of computer-generated random numbers	Yes (independent monitoring person)	Follicular development characteristics
Cédrin-Durnerin et al., 2006, France, Hum Reprod	Yes (six centers)	2004	49 (25 estradiol, 24 control)	Table of random numbers	Yes (sealed envelopes)	Follicular growth hormonal profiles
Blockeel et al., 2012, Belgium, RBM Online	No	2010–2011	86 (44 estradiol, 24 control)	Computer generated list	Yes (sealed envelopes)	Number of patients undergoing oocyte retrieval on weekends
Cédrin-Durnerin et al., 2012, France, Hum Reprod	Yes (ten centers)	2006–2010	472 (238 estradiol, 234 control)	Table of random numbers	Yes (sealed envelopes)	Number of retrieved oocytes
Nejad et al., 2018, Iran, Int J Reprod Biomed	No	Not provided	186 (53 OCP, 63 estradiol, 70 control)	Table of random numbers	Yes (independent monitoring person)	Number of mature oocytes, clinical pregnancy
Ye et al., 2009, China, J Assist Reprod Genet	No	2006–2007	220 (109 estradiol, 111 control)	Table of computer generated random numbers	Yes (sealed envelopes)	Clinical pregnancy, live birth rate, early pregnancy loss rate
Sefrioui, et al., 2019, Gynecol Endocrinol	No	2015–2018	244 (122 estradiol, 122 control)	Not provided	Not provided	Number of mature oocytes, clinical pregnancy rate, live birth rate

**Table 2:** Study population in the included RCTs

Study	Inclusion criteria	Exclusion criteria
Fanchin et al., 2003	Age <38 years; regular norm ovulatory cycles (25–35 days); BMI = 18–27 kg/m^2^; no hormone therapy during the past 6 weeks	Major protocol violation
Cédrin-Durnerin et al., 2006	Age <38 years; regular norm ovulatory cycles (28–35 days); BMI = 18–30 kg/m^2^	High levels of baseline FSH or E2, <5 follicles at the AFC performed on Day 3 of a spontaneous cycle or a history of high (>20 oocytes) or low (<5 oocytes) ovarian response in a previous IVF attempt
Blockeel et al., 2012	Age <36 years; regular norm ovulatory cycles (25–35 days); BMI = 18–29 kg/m^2^; Day 3 serum FSH concentration <12 IU/l; first or second IVF/ICSI cycle	Oocyte donors; endometriosis grade 3 or more; endocrine or metabolic abnormalities; polycystic ovary syndrome or a previous history of poor ovarian response
Cédrin-Durnerin et al., 2012	Age <38 years; regular norm ovulatory cycles (28–35 days); BMI = 18–30 kg/m^2^; first or second IVF/ICSI attempt	High basal levels of serum FSH (>12 IU/L) or E2 (>80 pg/mL); on Day 3 AFC ≤5; history of high (>20 oocytes) or low (<5 oocytes) ovarian response in an earlier IVF attempt
Shahrokh et al., 2018	Age 18–35 years; BMI = 19–30 kg/m^2^; ≤2 IVF attempts; AMH = 1–6 ng/mL; fresh embryo transfer	FSH >10 IU/I or AFC <4; hydrosalpinx; uterus disorders such as uterus fibroid endocrine disorder, and polycystic ovarian syndrome
Ye et al., 2009	Age 25–35 years; BMI = 18–25 kg/m^2^; previous IVF cycles <3; no previous poor response; normal ovulatory cycles (25–35 days); no hormone therapy within the past 3 months	Major protocol violation
Sefrioui, et al., 2019	Age <40 years; BMI = 18–30 kg/m^2^; Day 3 serum FSH concentration <12 IU/l, E2 <75 pg/mL; AMH = 1.3–2.6 ng/mL normo-ovulatory cycles (25–35 days); first or second IVF/ICSI attempt	Patients not meeting the inclusion criteria as well as those with known history of polycystic ovarian syndrome, endometriosis, previous high ovarian response, or repeated IVF failures

IVF = *in vitro* fertilization; AFC = antral follicle count; BMI = body mass index; ICSI = intracytoplasmic sperm injection; E2 = estrogen; AMH = anti-Mullerian hormone.

**Table 3:** Treatment protocols in the included RCTs

Study	Estrogen	Initiation/duration of estradiol pretreatment	Gonadotropin type/starting dose	GnRH antagonist initiation	Criteria for triggering	Luteal support
Fanchin et al., 2003	17β-estradiol 4 mg/day	Cycle Day 20–next cycle 2	r-FSH at a fixed dose of 225 IU/day	Cetrorelix by a leading follicle ≧13 mm	At least five follicles >16 mm	Intravaginal micronized progesterone 400 mg
Cédrin-Durnerin et al., 2006	17-β estradiol 2 mg twice a day	10 days before the presumed menses, for 10–15 days	r-FSH 150–300 IU/day	Ganirelix by a leading follicle ≧14 mm	At least three follicles > 17 mm	Intravaginal micronized progesterone 400 mg
Blockeel et al., 2012	Estradiol 2 mg twice a day	From cycle Day 25 onwards for 6–10 days	r-FSH 150 IU/day	Ganirelix fixed on Day 6	At least three follicles >17 mm	Intravaginal micronized progesterone 600 mg
Cédrin-Durnerin et al., 2012	17β-estradiol 2 mg twice a day	Started 7 days before the presumed menses	r-FSH 150 IU/day with a 50 IU increment when aged >35 years	Ganirelix fixed on Day 6	At least three follicles >17 mm	Intravaginal micronized progesterone 400 mg and 17β-estradiol 2 mg
Nejad et al., 2018	Estradiol valerate tablet 2 mg twice a day	From Day 20 of the previous cycle for 10 days	r-FSH 150 IU/day	Cetrotide by a leading follicle ≧13 mm	At least two follicles >17 mm	Intravaginal micronized progesterone 400 mg
Ye et al., 2009	Estradiol valerate tablet 4 mg/day	From Day 21 until Day 2 of next cycle	r-FSH 150 IU/day	Cetrotide by a leading follicle ≧12–14 mm	At least three follicles ≧18 mm	Progesterone 80 mg/day
Sefrioui, et al., 2019	17β-estradiol 4 mg/day	From Day 20 until Day 1 next cycle	r-FSH 150–300 IU/day	Ganirelix starting on Day 5 or Day 6	At least three follicles ≧17 mm	Intravaginal micronized progesterone 600 mg

r-FSH = recombinant follicle stimulating hormone.

None of the seven analyzed studies declared financial support by pharmaceutical companies. Live birth rate was reported in four studies [13,14,16,17]. None of these trials was powered sufficiently to detect significant differences in ongoing pregnancy rate between patients with and those without estradiol pretreatment (Tables 1–3).

The quality of the enrolled studies was evaluated by the Cochrane Collaboration tool (Figure 2). Three RCTs did not provide abortion rate or early pregnancy loss rate, which we considered a high risk of incomplete data and selective outcome reporting [11,15,17]. One RCT of a small group size was rated as high risk in other biased domains [11]. One RCT did not explain the random method, which was rated as having unclear risk of bias in random sequence generation domain [17].

### 3.2 Primary outcomes

#### 3.2.1 Ongoing pregnancy rate

**Figure 3:**
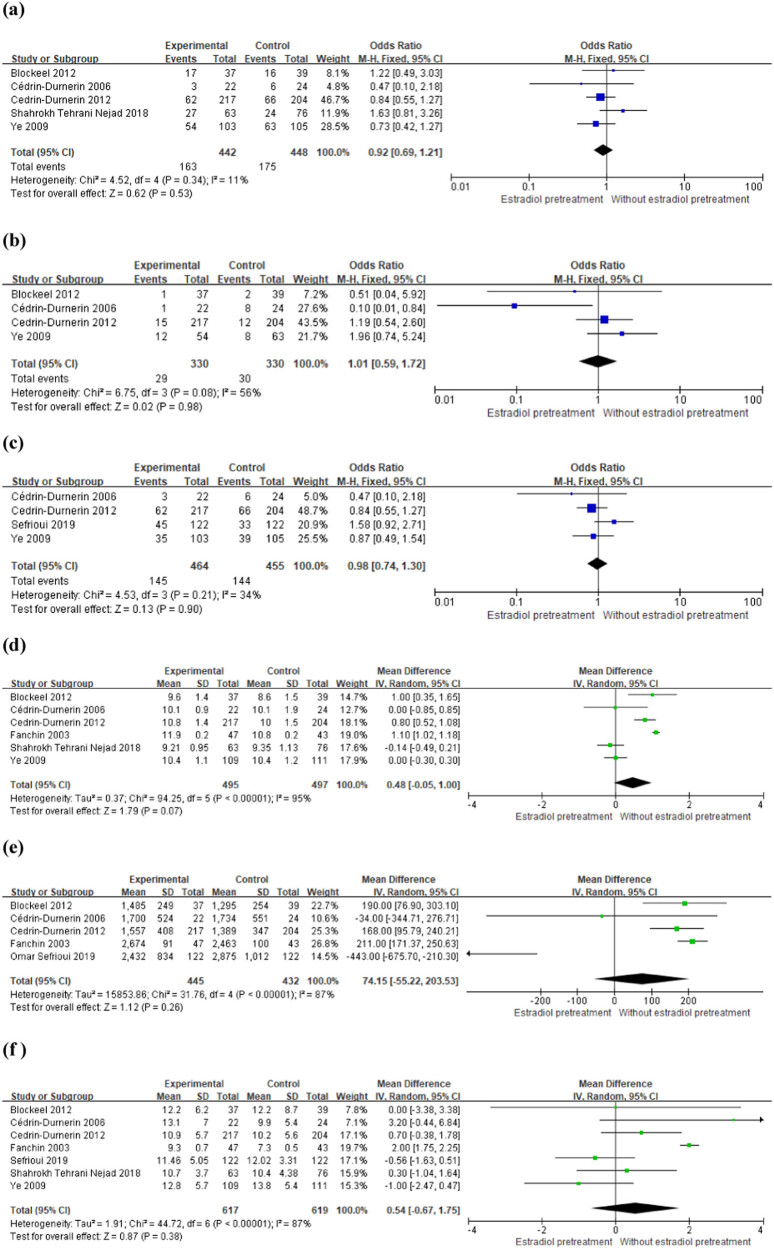
Forest plot of outcomes per cycle with or without estrogen pretreatment in women undergoing IVF/ICSI with GnRH antagonist protocol. Odds ratios and weighted mean differences for the outcomes: (a) ongoing pregnancy per cycle, (b) abortion rate, (c) live birth rate per cycle, (d) duration of gonadotropin exposure (days), (e) gonadotropin consumption (international units), and (f) number of cumulus–oocyte complexes. OR = odds ratio; WMD = weighted mean difference; CI = confidence interval; SD = standard deviation.

Five trials with a total of 890 women randomized provided data on the ongoing pregnancy rate [12–17]. The pooled analysis of the five trials did not present any significant differences between luteal estradiol pretreated patients and those without pretreatment as shown by OR: 0.92 (95% CI: 0.69–1.21; *P* = 0.53; heterogeneity: *P* = 0.34; fixed-effects model) (Figure 3a).

#### 3.2.2 Abortion rate

Four trials with a total of 660 women randomized provided data on the abortion rate [12,13,14,16]. The meta-analysis did not show any significant differences between patients with estradiol pretreatment and those without pretreatment, as shown by OR: 1.01 (95% CI: 0.59–1.72; *P* = 0.98; heterogeneity: *P* = 0.08; fixed-effects model) (Figure 3b).

#### 3.2.3 Live birth rate

Four trials with a total of 919 women randomized provided data on the live birth rate [13,14,16,17]. The data of the four trials also did not show any differences between patients with estradiol pretreatment and those without pretreatment, as shown by OR: 0.98 (95% CI: 0.74–1.30; *P* = 0.90; heterogeneity: *P* = 0.21; fixed-effects model) (Figure 3c).

### 3.3 Secondary outcomes

#### 3.3.1 Gonadotropin exposure time

The time of gonadotropin exposure was comparable between the estradiol pretreatment group and control group (Figure 3d). The combining data of the six trials provided support for this outcome via a random-effects model (WMD: +0.48 days, 95% CI: −0.55–1.00; *P* = 0.07).

#### 3.3.2 Gonadotropin consumption

No significant difference was detected in gonadotropin consumption between the estradiol pretreatment group and control group (Figure 3e). The combining data of the five trials provided support for this outcome via a random-effects model (WMD: 74.15 IU, 95% CI: −55.22–203.53; *P* = 0.26).

#### 3.3.3 Number of oocytes retrieved

No significant difference was found in number of oocytes retrieved between the two groups (Figure 3f) for which a random-effects model was used (WMD: 0.54, 95% CI: −0.67–1.75, *P* = 0.38).

## 4 Discussion

An ovarian stimulation protocol with GnRH antagonist shows great advantages in improving the patients’ experience and visit satisfaction of IVF/ICSI treatment [18,19]. It is therefore speculated that COH with GnRH antagonist protocols will be widely used over time [20]. The debate about the influence of estradiol pretreatment on reproductive outcomes in GnRH antagonist cycles, thus, remains a “hot topic.” In the present meta-analysis, we found that the available data based on comprehensive analysis of the enrolled RCTs did not show definite difference in ongoing pregnancy per cycle, abortion rate, and live birth rate per cycle between patients who were administrated estradiol pretreatment and those who were not before COH in GnRH antagonist protocol. Furthermore, no significant difference was detected in gonadotropin exposure time, gonadotropin consumption, and number of oocytes retrieved between the two groups (Figure 3). Possible reasons for the results are that all the patients in the seven included studies are with normal ovarian function. Patients with normal ovarian function can obtain sufficient oocytes during COH to counteract the negative effects of follicular desynchrony in the GnRH antagonist protocol. Therefore, in patients with normal ovarian function, it needs to be treated with caution if luteal estradiol pretreatment is only designed to improve follicle synchronization. However, viewed from another angle, the data of the present meta-analysis demonstrate that luteal phase estrogen pretreatment has no negative effect on pregnancy outcomes. Therefore, it can be used for cycle scheduling in patients with normal ovarian function when the GnRH antagonist protocol is used in IVF/ICSI treatment. Cycle scheduling has become very important for GnRH antagonist protocol since the start of the cycle depends on the menstruation occurrence. Luteal estrogen pretreatment schedule menstrual cycle by inhibiting the increase of spontaneous FSH in patients and affect follicle growth by regulating spontaneous and exogenous FSH in the whole process of COH. Optimal cycle scheduling can be applied to avoid oocyte retrieval during weekends and to optimize the allocation for week-intensive workloads. The temperature, gas concentration, and humidity inside the incubator thereby could be kept stable by avoiding excessive opening and closing of the doors. Cycle scheduling can reduce unplanned work and increase concentration and efficiency of the laboratory staff [21,22]. Besides, it is also very convenient for patients, as it offers flexibility in timing the start of ovary stimulation. The most common treatments for cycle scheduling are OCs and estrogen. The OCs may induce a negative effect on endometrial receptivity in the starting cycle and decrease the embryo implantation rate. However, estrogen is considered the optimal choice for cycle scheduling because it exhibits no negative effect on endometrium. As a result, estrogen would be widely used to regulate the initiation of ovarian stimulation and the timing of oocytes retrieval in patients.

Despite there was significant heterogeneity between the seven individual trials for the secondary outcome, and all data have shown the same effect of estradiol administration on the gonadotropin exposure time and gonadotropin consumption. In the current study, estradiol pretreatment does not increase the gonadotropin exposure time and gonadotropin consumption in patients with normal ovary response in a GnRH antagonist protocol. It is well known that a GnRH antagonist protocol offered an advantage of shorter duration of ovarian stimulation over agonist protocol [20]. Thus, comparison between an estradiol pretreatment GnRH antagonist and a long agonist protocol relating to this view would be meaningful. In Ye et al.’s study [16], it was shown that there were no significant differences in duration of stimulation and dosage of gonadotropin between GnRH antagonist protocol with estradiol pretreatment and standard long GnRH agonist protocol. However, an estradiol pretreatment GnRH antagonist protocol might offer several practical and theoretical advantages over a long GnRH agonist protocol because estradiol pretreatment begins in the midluteal phase, a scheduling GnRH antagonist protocol for IVF/ICSI can be started in the same menstrual cycle even if the patients express a temporary treatment decision after the early follicular phase. Furthermore, they have gained one more chance to achieve pregnancy spontaneously before cycle starting and the cycle could be scheduled based on the clinical and embryological laboratory working arrangement.

The present study displayed the following advantages in terms of research methods. To reduce the risk of bias, only RCTs were included in the meta-analysis. The included trials were assessed according to the modified Jadad scale (Jadad score ≥3). To minimize potential bias in the overview process, we used more than two reviewers in the literature screening, data extraction, and quality assessment. However, there are still some limitations in the present study. First, the data of the RCTs were not all available for pooled analysis, although we had tried our best to contact the authors. Therefore, the ongoing pregnancy rate was used as a surrogate outcome measure instead of live birth rate in some of the included trials. Second, the heterogeneity related to the secondary outcome, limited the overall persuasiveness of the results. Because of the insufficient studies included, subgroup analyses or sensitivity analyses failed to explore the source of heterogeneity. As a result, the evidence of combining data has been limited. The potential sources of heterogeneity are expected to be partly due to patients from different studies and partly due to different patient baseline characteristics. Therefore, there is an urgent need for further, larger sized RCTs relating to estradiol pretreatment in GnRH antagonist protocols. This will produce a more definite assessment of effects on reproductive outcomes when estradiol pretreatment is administrated. Ideally, besides ongoing pregnancy, live birth should also be included in primary outcomes in such future studies.

In conclusion, luteal estradiol pretreatment in IVF/ICSI cycles with GnRH antagonist protocol in normal ovary responding population did not affect the reproductive outcomes and increase the duration and gonadotropin consumption in COH. Therefore, luteal estradiol pretreatment is an encouraging option to facilitate cycle scheduling in GnRH antagonist protocol. Cycle scheduling with luteal estradiol pretreatment can be used to schedule oocyte retrieving time, avoiding weekend retrievals, and reducing the amount of unplanned work, which can result in loss of concentration and reduced efficiency of the laboratory staff. The present study could provide reference data for clinicians in the field of assisted reproduction in programing an COH protocol. However, further prospective studies are necessary to draw more firm conclusions on pregnancy likelihood following estradiol pretreatment before ovarian stimulation.
